# Estimating the relative rates of lipopolysaccharide synthesis in *Escherichia coli* K-12 by click chemistry-mediated labeling

**DOI:** 10.1371/journal.pone.0325589

**Published:** 2025-06-23

**Authors:** Sheng Shu, Wei Mi

**Affiliations:** 1 Department of Pharmacology, Yale University School of Medicine, New Haven, Connecticut, United States of America; 2 Department of Molecular Biophysics and Biochemistry, Yale University, New Haven, Connecticut, United States of America; 3 Department of Microbial Pathogenesis, Yale University School of Medicine, New Haven, Connecticut, United States of America; Purdue University, UNITED STATES OF AMERICA

## Abstract

Lipopolysaccharide (LPS), a critical glycolipid component of Gram-negative bacteria, plays a central role in bacterial membrane integrity and host immune interactions. Despite extensive studies on the regulation of LPS synthesis, methods to quantify its synthesis rate remain limited. Here, we present a novel approach to measure *in vivo* LPS synthesis rates in the *E. coli* K-12 strain MG1655 using click chemistry. This method involves the incorporation of an exogenous Kdo analog, 8-azido-3,8-dideoxy-D-manno-oct-2-ulosonic acid (Kdo-N_3_), into newly synthesized LPS, followed by a copper-free click reaction with a fluorescent alkyne. The labeled LPS is separated by SDS-PAGE and visualized via in-gel fluorescence. We compared two fluorescent dyes and found that AZDye 488 DBCO exhibited stronger sensitivity for labeling LPS during the log phase of bacterial growth. Our results further demonstrated that the amount of newly synthesized LPS correlates linearly with the pulse labeling time of Kdo-N_3_, validating this approach as a reliable method for estimating relative LPS synthesis rates during the exponential phase of *E. coli* MG1655 growth. This method offers a reliable, non-radioactive approach for measuring LPS synthesis *in vivo*, providing a powerful tool to investigate bacterial physiology and the regulation of LPS biogenesis.

## Introduction

Lipopolysaccharide (LPS) is a glycolipid located in the outer membrane of Gram-negative bacteria [1], playing a crucial role in both their structural integrity [2,3] and interaction with the host immune system [4]. LPS consists of three main components: lipid A, which anchors the molecule into the bacterial membrane and is responsible for its toxic properties; the core polysaccharide, which links lipid A through the Kdo (3-deoxy-D-manno-octulosonic acid) saccharide [4]; and the O-antigen (OAg), a highly variable polysaccharide chain that extends outward from the bacterium.

LPS labeling is a technique used to study the structure, distribution, and synthesis of LPS in Gram-negative bacteria. Traditionally, radioactive labeling methods have been employed, using orthophosphate (^32^P) or sodium [1-^14^C]acetate [5,6,7]. However, these methods can inadvertently label other phospholipids or fatty acids alongside LPS. Additionally, the use of radioactive isotopes presents significant safety concerns, requiring specialized equipment and radiation safety facilities [8], which limits accessibility and makes it less feasible for some research labs.

Because Kdo is a conserved and essential component of the inner core of LPS [9,10] and is present in the LPS of nearly all Gram-negative species [11], metabolic incorporation of clickable carbohydrates has been has been developed recently as a highly specific method for labeling LPS. This approach uses an exogenous Kdo analog, 8-azido-3,8-dideoxy-D-manno-oct-2-ulosonic acid (Kdo-N_3_), to metabolically label LPS [9,12–18]. Following a click reaction with a fluorescent alkyne, the labeled LPS can be visualized on the cell surface using light microscopy to analyze the localization of newly synthesized LPS molecules. Due to its specificity, click chemistry-mediated LPS labeling has gained popularity for tracking LPS localization in various studies [19,20]. However, previous applications have been limited to imaging. In this study, we investigate whether this method can be adapted for kinetic analysis and introduce a novel protocol to measure relative LPS synthesis rates in the *E. coli* K-12 strain MG1655. This advancement provides a powerful tool for monitoring a key parameter—*in vivo* LPS synthesis rates—in study LPS biogenesis.

## Materials and methods

The protocol described in this peer-reviewed article is published on protocols.io, DOI: dx.doi.org/10.17504/protocols.io.n92ld5bzov5b/v1 and is included for printing as S1 File with this article.

## Expected results

### Labeling newly synthesized LPS using click chemistry

**Fig 1:**
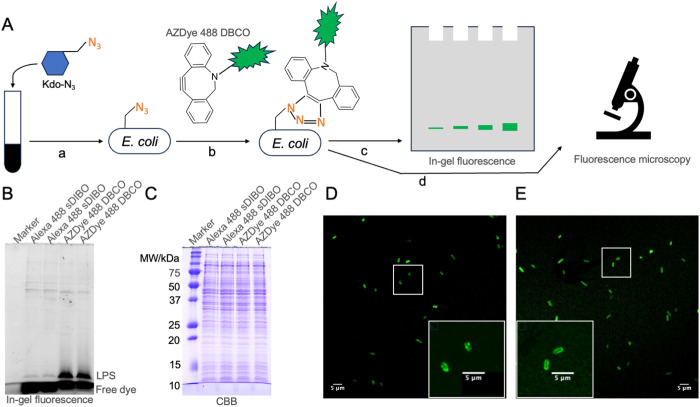
Labeling newly synthesized LPS using click chemistry. **(A)** Schematic of Kdo-N3 metabolic labeling: a) incubation of *E. coli* K-12 culture with Kdo-N_3_; b) copper-free click labeling with AZDye 488 DBCO; c) cell lysates are separated by SDS-PAGE, and the labeled LPS can be detected by in-gel fluorescence; d) the labeled cells can be visualized using fluorescence microscopy. **(B)** In-gel fluorescence of LPS labeled by two different fluorescent alkynes. For each fluorescent alkyne, duplicate labeling was conducted. **(C)** The same gel of **(B)** was stained with Coomassie Brilliant Blue R-250 (CBB). **(D, E)** Fluorescence microscope images of *E. coli* MG1655 cells incubated with 1 mM Kdo-N_3_ for 15 minutes (D) and 90 minutes **(E)**, followed by AZDye 488 DBCO labeling for 45 minutes. The boxed areas are zoomed in for greater detail.

To label newly synthesized LPS, we cultured the *E. coli* K-12 strain MG1655 in the presence of Kdo-N_3_ (Fig 1A). As previously described, Kdo-N_3_ is transported to bacterial cytosol, where it is processed and incorporated into newly synthesized LPS [9,14]. The LPS is then translocated from the inner membrane’s inner leaflet to the periplasmic leaflet by the flipase MsbA [21,22] and is subsequently transported to the cell surface via the lipopolysaccharide transport (Lpt) pathway [23,24,25]. A copper-free click reaction between the azide group on Kdo-N_3_ and a fluorescent alkyne allows the labeling of the newly synthesized LPS. After cell lysis, fluorescence-labeled LPS can be separated by SDS-PAGE and quantified by in-gel fluorescence. Additionally, the labeled LPS can be visualized using a fluorescence microscope (Fig 1A).

Different fluorescent alkynes exhibit varying labeling efficiencies in click chemistry-mediated LPS labeling [26]. To compare two commercially available fluorescent alkynes, we tested Alexa Fluor 488 sDIBO (Thermo Fisher Scientific C20020) and AZDye 488 DBCO (Vector Laboratories CCT-1278). Both alkynes feature similar fluorophores, with excitation and emission wavelengths of approximately 490 nm and 520 nm, respectively. We incubated the *E. coli* K-12 strain MG1655 in log phase with 1 mM Kdo-N_3_ for 30 minutes, then washed the cells with M9 minimal medium supplemented with maltose (M9-maltose) to slow cell growth during labeling [26]. The cells were then divided and labeled with 0.1 mM of either Alexa Fluor 488 sDIBO or AZDye 488 DBCO for 60 minutes. After washing the cells three times with M9-maltose, the lysates were separated by 15% SDS-PAGE, and the labeled LPS were visualized by in-gel fluorescence. Compared to Alexa Fluor 488 sDIBO, AZDye 488 DBCO resulted in a LPS signal approximately ten times stronger (Fig 1B), despite similar loading, as shown by Coomassie Brilliant Blue (CBB) staining in Fig 1C. Based on these results, we chose AZDye 488 DBCO for subsequent analysis.

To assess the feasibility of adapting this click chemistry labeling method for measuring *in vivo* LPS synthesis rates, we incubated the *E. coli* K-12 strain MG1655 with 1 mM Kdo-N_3_ for either 15 minutes or 90 minutes. Following incubation with 0.1 mM of AZDye 488 DBCO for 45 min, fluorescence microscopy was used to examine the cells. After 15 minutes of incubation with Kdo-N_3_, we observed fluorescent foci on the cell surface (Fig 1D). After 90 minutes, the fluorescent signal was more evenly distributed across the cell surface (Fig 1E). These findings are consistent with previous studies, which suggest that during cell growth, newly synthesized LPS is initially inserted at specific locations on the cell surface, rather than being randomly distributed [27,28,29,30]. Furthermore, this process takes time to fully distribute the LPS across the surface of the cell. Collectively, these results confirm that we successfully labeled newly synthesized LPS *in vivo* using click chemistry.

### Measuring the relative *in vivo* LPS synthesis rate using click chemistry

**Fig 2:**
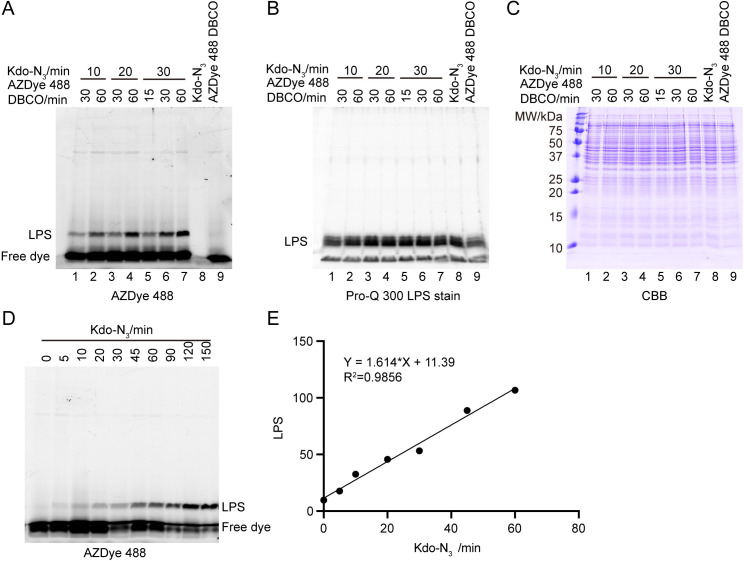
Optimization of click chemistry labeling conditions for LPS synthesis rate measurement. **(A)** In-gel fluorescence of click chemistry-labeled LPS from *E. coli* MG1655 incubated with 1 mM Kdo-N_3_ and 50 mM AZDye 488 DBCO for varying durations. Cell lysates were separated by 15% SDS-PAGE, and AZDye 488 signals were detected using the Bio-Rad ChemiDoc MP Imaging System. The bands corresponding to labeled LPS and free dye are indicated. **(B)** The same gel as in **(A)**, stained with Pro-Q™ Emerald 300 Lipopolysaccharide Gel Stain Kit according to the manufacturer’s instructions. The LPS bands are indicated. **(C)** The same gel as in **(B)**, further stained with CBB. **(D)** In-gel fluorescence of click chemistry-labeled LPS from log-phase *E. coli* MG1655 cells incubated with Kdo-N_3_ for varying durations. Cell lysates were separated by 15% SDS-PAGE, and AZDye 488 signals were scanned using the Bio-Rad ChemiDoc MP Imaging System. The labeled LPS and free dye bands are indicated. **(E)** Quantification of LPS band intensity from **(D)**, plotted against the incubation time with Kdo-N_3_. The linear regression equation and the corresponding R-squared value are shown.

To measure the *in vivo* LPS synthesis rate, it is essential to determine how much LPS is synthesized within a specific time frame. To minimize errors in measuring LPS synthesis rates caused by variations in Kdo-N_3_ incorporation efficiency, we aimed to use the shortest possible incubation time with Kdo-N_3_ while ensuring a reliable measurement signal. We therefore tested various incubation times with 1 mM Kdo-N_3_ for *E. coli* MG1655 during the logarithmic phase: 10, 20, and 30 minutes. Since the labeling efficiency is also influenced by the incubation time with fluorescent alkyne [14], we also optimized the incubation times with AZDye 488 DBCO. Specifically, we tested incubation times of 30 and 60 minutes for the 10- and 20-minute Kdo-N_3_ samples, and 15, 30, and 60 minutes for the 30-minute Kdo-N_3_ samples. The cell lysates were separated by 15% SDS-PAGE, and the AZDye 488-labeled LPS were visualized using in-gel fluorescence (Fig 2A). As expected, the cell sample grown with Kdo-N_3_ but without treatment with AZDye 488 DBCO showed no fluorescent bands (Fig 2A, lane 8). In contrast, the sample growing without Kdo-N3 but treated with AZDye 488 DBCO displayed a fluorescent band, likely corresponding to free dye that had permeated the cell during incubation (Fig 2A, lane 9). The samples from cells grown with Kdo-N_3_ and treated with AZDye 488 DBCO showed two distinct bands: the lower band, which may correspond to free dye or free Kdo-N3 labeled with AZDye 488 DBCO, and the upper band, which corresponds to newly synthesized LPS labeled with AZDye 488 DBCO (Fig 2A, lanes 1–7). By comparing the samples with different Kdo-N_3_ incubation, we observed that when fixing the AZDye 488 DBCO incubation time, longer Kdo-N_3_ incubation resulted in a stronger LPS band (Fig 2A, lanes 1, 3, 6 and lanes 2, 4, 7). Similarly, when fixing the Kdo-N_3_ incubation time, longer AZDye 488 DBCO incubation led to stronger LPS bands (Fig 2A, lanes 1–2, lanes 3–4, lanes 5–7). Fig 2B shows the same gel stained with the Pro-Q™ Emerald 300 Lipopolysaccharide Gel Stain Kit, indicating similar total LPS in all samples, while Fig 2C presents the same gel further stained with CBB, demonstrating comparable total protein levels. These data suggest that a 10-minute Kdo-N_3_ incubation followed by 30 minutes of AZDye 488 DBCO labeling is optimal for measuring LPS synthesis, as it provides a short Kdo-N_3_ labeling period while still yielding a clear staining band.

To validate this method for measuring *in vivo* LPS synthesis rates, we incubated log-phase *E. coli* MG1655 grown in M9 medium with Kdo-N_3_ for various durations. After incubation, the cells were treated with AZDye 488 DBCO for 30 minutes at 37°C. The cell lysates were separated by 15% SDS-PAGE, and the AZDye 488-labeled LPS were visualized using in-gel fluorescence (Fig 2D). When we quantified the amount of newly synthesized LPS and plotted it against the incubation time with Kdo-N_3_, we observed a linear relationship within the first 60 minutes (Fig 2E). This result suggests that *E. coli* cells synthesize LPS at a constant rate during the log phase. Overall, these data confirm that our method provides a reliable means to measure the *in vivo* LPS synthesis rate in *E. coli* K-12.

## Discussion

This article describes a method that uses click chemistry-mediated labeling to measure the LPS synthesis rate in *E. coli* K-12. In this procedure, Kdo-N_3_ is added to the cell culture for 10 minutes (Fig 1A(a)). After removing excess Kdo-N_3_ from the medium, the cells are incubated with AZDye 488 DBCO for 30 minutes (Fig 1A(b)). Afterwards, any unreacted AZDye 488 DBCO is washed away. The whole-cell fluorescent signal alone cannot be used to estimate the amount of newly synthesized LPS, as a significant amount of free dye is taken up by the cells or nonspecific binding of the dye to the cell surface. (Fig 2A lane 9). To accurately measure the LPS synthesis rate, it is necessary to separate labeled LPS from the free dye. Therefore, cell lysates are separated by SDS-PAGE, the LPS synthesis rate is quantified by measuring the in-gel fluorescence signal of the newly labeled LPS (Fig 1A(c)). The strong linear relationship between fluorescent intensity and Kdo-N_3_ incubation time supports the method’s accuracy in estimating the LPS synthesis rate.

One advantage of this method is that it does not require extraction of LPS from cells. Instead, following Kdo-N₃ incorporation and click chemistry labeling, whole-cell lysates are directly separated on a 15% SDS-PAGE gel. In Fig 2A, we observed a single visible band corresponding to newly synthesized LPS, whereas Fig 2B shows multiple bands representing total LPS. This difference arises from the fact that LPS molecules exist in various stages of synthesis and transport. As a result, the total LPS pool includes both fully assembled and partially assembled core structures; not all LPS molecules have a complete core. The high-percentage gel (15%) provides sufficient resolution to separate LPS species based on differences in core completeness, resulting in the multiple bands seen in Fig 2B. In contrast, the in-gel fluorescence signal in Fig 2A corresponds specifically to newly synthesized LPS labeled with AZDye 488. Due to the limited membrane permeability of AZDye 488 DBCO, the dye primarily reacts with Kdo-N₃-labeled LPS present on the cell surface. These surface-exposed LPS species are mostly those with fully assembled cores, leading to the appearance of a single visible band in the fluorescence image.

Compared with radioactive labeling of LPS, this method allows experiments to be conducted using standard laboratory equipment without the need for radiation safety facilities, making it accessible to most laboratories. Another advantage is that it eliminates the need to extract lipid molecules and separate LPS from other phospholipids, significantly reducing the time required to measure LPS synthesis rates compared to traditional radioactive labeling methods. Given that Kdo is an essential component widely present in LPS across Gram-negative bacteria, the method described here has the potential to be developed as a general tool for estimating LPS synthesis rates in a broad range of bacterial species. However, several parameters must be considered when adapting this method to other bacteria, including the efficiency of Kdo-N_3_ uptake, optimization of click chemistry labeling efficiency, and accurate quantification of the fluorescent signal for smooth LPS. Moreover, it is important to consider the presence of other Kdo-containing glycoconjugates. For instance, many Gram-negative bacteria produce capsular polysaccharides (CPS) [31,32], and among the four recognized CPS groups, groups 2 and 3 are known to contain Kdo [33,34]. However, *E. coli* K-12 strains, including MG1655 used in our study, produce only minimal amounts of CPS under standard laboratory conditions [35]. As such, we believe that the contribution of these glycoconjugates to overall Kdo-N₃ incorporation is negligible and does not significantly affect the specificity of LPS labeling in our assay.

This method provides only a relative measure of LPS synthesis rates, because we lack a standard curve to determine the absolute quantity of LPS based on fluorescent signal intensity, and we cannot quantify the incorporation efficiency of Kdo-N3 into LPS. Nevertheless, it remains highly useful for comparing LPS synthesis rates under different growth conditions during the exponential phase, such as assessing the effects of various antibiotics on LPS synthesis and bacterial growth rates.

## Supporting information

S1 File Step-by-step protocol, also available on protocols.io. (PDF)
