# Supplementary material for: Estimating the relative rates of lipopolysaccharide synthesis in Escherichia coli K-12 by click chemistry-mediated labeling
Source: PLoS One. 2025 Jun 23;20(6):e0325589. doi: 10.1371/journal.pone.0325589 (PMC12184891; doi:10.1371/journal.pone.0325589)
Supplement: S1 File — (PDF) [file pone.0325589.s001.pdf]

# Estimating the Relative Rates of Lipopolysaccharide Synthesis in *Escherichia coli* K-12 by Click Chemistry-Mediated Labeling

RESERVED DOI:
10.17504/protocols.io.n92ld5bzov5b/v1

Sheng Shu[1,2], Wei Mi[1,2]

[1]Department of Pharmacology, Yale University School of Medicine, New Haven, CT 06520, USA;
[2]Department of Molecular Biophysics and Biochemistry, Yale University, New Haven, CT 06520, USA

Sheng Shu
Yale University

**Protocol Info:** Sheng Shu, Wei Mi . Estimating the Relative Rates of Lipopolysaccharide Synthesis in Escherichia coli K-12 by Click Chemistry-Mediated Labeling. **protocols.io** **https://protocols.io/view/estimating-the-relative-rates-of-lipopolysaccharid-d5ux86xn**

**Created:** March 13, 2025

**Last Modified:** March 17, 2025

**Protocol Integer ID:** 124535

**Keywords:** Lipopolysaccharide, LPS, Click-chemistry, Kdo, in-gel fluorescence, E. coli

**Funders Acknowledgements:**
**Wei Mi**
Grant ID: R01GM137068
**Wei Mi**
Grant ID: RM1GM149406

## Abstract

Lipopolysaccharide (LPS), a critical glycolipid component of Gram-negative bacteria, plays a central role in bacterial membrane integrity and host immune interactions. Despite extensive studies on the regulation of LPS synthesis, methods to quantify its synthesis rate remain limited. Here, we present a novel approach to measure in vivo LPS synthesis rates in E. coli K-12 strain MG1655 using click chemistry. This method involves the incorporation of an exogenous Kdo analog, 8-azido-3,8-dideoxy-D-manno-oct-2-ulosonic acid (Kdo-N3), into newly synthesized LPS, followed by a copper-free click reaction with a fluorescent alkyne (AZDye 488 DBCO). The labeled LPS is separated by SDS-PAGE and visualized via in-gel fluorescence. We optimized the labeling conditions by testing different incubation times for Kdo-N3 and AZDye 488 DBCO, ultimately identifying a 10-minute Kdo-N3 incubation and 30-minute AZDye 488 DBCO labeling as optimal for quantifying LPS synthesis. Our results demonstrate that the amount of newly synthesized LPS correlates linearly with incubation time, particularly during the log phase of bacterial growth. This method offers a reliable, non-radioactive approach for measuring LPS synthesis in real-time, providing valuable insights into bacterial physiology and the regulation of LPS biogenesis.

# Materials

## Materials

1. *E. coli* K-12 strain MG1655: The Coli Genetic Stock Center at Yale University
2. Culture tube: Falcon, catalog number: 352059
3. 250 ml flask: Pyrex, catalog number: 4980
4. 15 ml centrifuge tube: Falcon, catalog number 352096
5. 1.5 ml microcentrifuge tube: USA Scientific, catalog number: 1615-5500
6. 0.2 µm syringe filter: Pall, catalog number: 4602
7. Pro-Q Emerald 300 LPS staining kit: Thermo Fisher Scientific, Catalog number: P20495
8. 15% SDS-PAGE gel: homemade

## Recipe

**M9-glucose minimal medium**: dissolve 1.13 g M9 minimal medium salts (MP biomedicals, catalog number: 3037032) in 100 mL of Milli-Q water. After autoclave, add 200 µL of [M] 1 Molarity (M) $MgSO_4$ (Sigma, catalog number: 208094) (sterilized by autoclave), 10 µL of [M] 1 Molarity (M) $CaCl_2$ (J.T. Baker, catalog number: 1332-1) (sterilized by autoclave) and 1 mL of 40% glucose (Sigma, catalog number: G7021) (sterilized by filtering).

| A | B |
|---|---|
| M9 minimal medium salts | 1.13 g |
| Milli-Q water | 100 ml |
| 1M $MgSO_4$ | 200 µl |
| 1M $CaCl_2$ | 10 µl |
| 40% glucose | 1 ml |

**M9-maltose minimal medium**: Dissolve 1.13 g M9 minimal medium salts (MP biomedicals, catalog number: 3037032) in 100 mL of Milli-Q water. After autoclave, add 200 µL of [M] 1 Molarity (M) $MgSO_4$ (Sigma, catalog number: 208094) (sterilized by autoclave), 10 µL of [M] 1 Molarity (M) $CaCl_2$ (J.T. Baker, catalog number: 1332-1) (sterilized by autoclave) and 1 mL of 20% maltose (Sigma, catalog number: M5895) (sterilized by filtering).

| A | B |
|---|---|
| M9 minimal medium salts | 1.13 g |
| Milli-Q water | 100 ml |
| 1M $MgSO_4$ | 200 µl |
| 1M $CaCl_2$ | 10 µl |

| A | B |
| --- | --- |
| 20% maltose | 1 ml |

**Kdo-$N_3$ Stock solution** ( [M] 0.5 Molarity (M) ): Dissolve 100 mg of Kdo-$N_3$ (Vector Laboratories, catalog number CCT-1241-100) in 714 µL of Milli-Q water. Filter the stock solution using a 0.2 µm syringe filter, aliquot to 20 µL in 1.5 ml microcentrifuge tubes, and store at -20 °C .

| A | B |
| --- | --- |
| Kdo-N3 | 100 mg |
| Milli-Q water | 714 µl |

**AZDye 488 DBCO stock solution** ( [M] 10 millimolar (mM) ): Dissolve 5 mg of AZDye 488 DBCO (Vector Laboratories, catalog number CCT-1278) in 631 µL of DMSO. Aliquot to 20 µL in 1.5 ml microcentrifuge tubes, and store at -80 °C .

| A | B |
| --- | --- |
| AZDye 488 DBCO | 5 mg |
| DMSO | 631 µl |

**Lysis buffer**:

| A | B |
| --- | --- |
| Tris-HCl, pH 7.8 | 50 mM |
| Nacl | 300 mM |
| Glycerol | 10 |

**4×SDS loading buffer**:

| A | B |
| --- | --- |
| Tris-HCl (pH 6.8) | 0.25M |
| SDS | 8% |
| Glycerol | 40% |
| Bromophenol blue | 0.02% |

**SDS running buffer**:

| A | B |
|---|---|
| Tris base | 25 mM |
| Glycine | 192 mM |
| SDS | 0.10% |

**Coomassie Blue stain solution**:

| A | B |
|---|---|
| Coomassie R-250 | 0.10% |
| Methanol | 40% |
| Acetic acid | 10% |

**Coomassie Blue destain solution**:

| | |
|---|---|
| Ethanol | 30% |
| Acetic acid | 10% |

**Equipment**

1. Incubator shaker: any brand
2. Nanodrop spectrophotometer: Thermo Scientific, Catalog Number:13-400-525
3. Milli-Q water system: Millipore Milli-Q Reference A+
4. Microcentrifuge: Thermo Scientific, Sorvall Legend Micro 21
5. Centrifuge: Eppendorf model number 5810R
6. Sonication: BRANSON Sonifier 250
7. heating block: Benchmark BSH1002
8. Power source: Bio-Rad, PowerPac Basic
9. Gel running apparatus: Bio-Rad, Mini Protean Tetra Cell
10. Imager: Bio-Rad, ChemiDoc MP imager
11. Platform shaker: Benchmark, Orbi Shaker JR.
12. Scanner: Epson Perfection V600 Photo

Corning™ Falcon™Round-Bottom Polypropylene Tubes **Fischer Scientific Catalog #**352059

PYREX® 250 mL Narrow Mouth Erlenmeyer Flasks with Heavy Duty Rim **Corning Catalog #**4980-250

15mLI polypropylene centrifuge tubes **Corning Catalog #**352096

- Seal-Rite® 1.5 mL Microcentrifuge Tubes **USA Scientific Catalog** #1615-5500
- Acrodisc Syringe Filters with Supor Membrane, Sterile - 0.2um, 13mm, 75/pk **Tisch Scientific Catalog** #Pall 4602
- Pro-Q™ Emerald 300 Lipopolysaccharide Gel Stain Kit **Thermo Fisher Catalog** #P20495

- M9 minimal medium salts **MP Biomedicals Catalog** #113037012-CF
- Magnesium sulfate **Merck MilliporeSigma (Sigma-Aldrich) Catalog** #208094
- JT Baker® 1332-01 Calcium Chloride, Dihydrate, Granular, BAKER ANALYZED™ ACS Reagent Grade, 500g Pol **Capitol Scientific Catalog** #JTB-1332-01
- D-( )-Glucose **Merck MilliporeSigma (Sigma-Aldrich) Catalog** #G7021
- D-(+)-Maltose monohydrate **Merck MilliporeSigma (Sigma-Aldrich) Catalog** #M5895
- Kdo Azide **Vector Laboratories Catalog** #CCT-1241
- AZDye 488 DBCO **Vector Laboratories Catalog** #CCT-1278

### Equipment

| | |
|---|---|
| Nanodrop™ One Spectrophotometer with WiFi and Qubit™ 4 Fluorometer | NAME |
| Spectrophotometer | TYPE |
| Thermo Scientific™ | BRAND |
| 13-400-525 | SKU |
| https://www.fishersci.com/shop/products/nanodrop-one-spectrophotometer-wifi-qubit-4-fluorometer/13400525 | LINK |

## Kdo-N3 Incorporation into *E. coli* K-12 Cells

1 Inoculate a single colony of *E. coli* K-12 MG1655 strain into a culture tube containing 3 mL of M9-glucose medium. Grow the culture Overnight in a incubator shaker at 220 rpm, 37°C .

2 The following day, measure the optical density (OD) at 600 nm (OD600) using a Nanodrop spectrophotometer. Dilute the overnight culture into 50 mL of fresh M9-glucose medium in a 250 mL flask to achieve a starting OD600 of 0.05.

3 Allow the culture to grow in the incubator shaker at 220 rpm, 37°C until the OD600 reaches approximately 0.35 (log phase).

4 Transfer 2 mL of the log-phase culture to a new culture tube and add 4 µL of Kdo-$N_3$ stock solution ( [M] 0.5 Molarity (M) ). This will give a final Kdo-$N_3$ concentration of [M] 1 millimolar (mM) . Incubate the culture at  in the shaker at 220 rpm, 37°C, 00:10:00 .

## Click-chemistry to label Kdo-N3

40m

5 Transfer the samples to a 15 mL centrifuge tube and centrifuge at 2000 x g, Room temperature, 00:04:00 . Discard the supernatant and gently wash the cell pellets with 1 mL of M9-maltose medium by pipetting.

4m

6 Centrifuge the cells again at 2000 x g, Room temperature, 00:04:00 . Discard the supernatant and resuspend the cell pellet in 50 µL of M9-maltose medium.

4m

7 Transfer the resuspended cells to a sterile 1.5 mL microcentrifuge tube. Add 0.5 µL of AZDye 488 DBCO stock solution ( [M] 10 millimolar (mM) ) to achieve a final concentration of [M] 0.1 millimolar (mM) AZDye 488 DBCO. Wrap the microcentrifuge tube with aluminum foil and incubate it in a incubator shaker at 220 rpm, 37°C, 00:30:00 .

30m

8 After incubation, centrifuge the cells at 4500 x g, Room temperature, 00:02:00 using a microcentrifuge. Discard the supernatant and gently wash the cell pellet with 1 mL of M9-maltose medium by pipetting.

2m

9 Repeat step 8 to wash the cells two more times.

10 After the final wash, remove the wash medium and resuspend the cell pellets in 200 µL of lysis buffer.

## SDS-PAGE and In-gel fluorescence

30m

11 Lyse the *E. coli* cells using a sonicator. After cell lysis, measure the absorbance at 600 nm using a Nanodrop spectrophotometer.

12 Nanodrop spectrophotometer to determine protein concentration.

13 Mix 30 µL of cell lysate with 10 µL of 4X SDS loading buffer. Boil the samples at 95 °C for 00:10:00 using a heating block.

10m

14 Load (50/ A280) µL of the prepared sample onto an SDS-PAGE gel. Run the gel at 180 V until the dye front reaches the bottom.

15 After electrophoresis, briefly rinse the gel with Milli-Q water. Capture an image of the gel using a Bio-Rad ChemiDoc MP imager.

16 Quantify the density of the LPS bands using ImageJ software. This will provide an estimate of the LPS synthesis rate.

## Staining of the total LPS

17 After checking the in-gel fluorescence of AZDye 488, stain the gel for total LPS using the Pro-Q Emerald 300 LPS Staining Kit, following the manufacturer’s instructions.

18 Capture an image of the Pro-Q Emerald 300 fluorescence signal using the Bio-Rad ChemiDoc MP imager.

## Staining of the total Protein

1h

19 After visualizing the Pro-Q Emerald 300 signal for total LPS, stain the gel for total protein by incubating it in 50 mL of Coomassie Blue stain solution at Room temperature on a platform shaker set to 60 rpm, 00:20:00 .

20m

20 Discard the Coomassie Blue stain solution and rinse the gel twice with tap water.

21 Add 50 mL of Coomassie Blue destain solution and incubate the gel on a platform shaker at 60 rpm, 00:20:00 . 20m

22 Repeat Step 21 two to three times, or until the background is sufficiently clear.

23 Wash the gel with 50 mL of tap water for 00:20:00 to remove excess stain. 20m

24 Scan the gel using a scanner to capture the image.
